# Flower power: Modeling floral resources of wild cherry (*Prunus avium* L.) for bee pollinators based on 3D data

**DOI:** 10.1002/ecy.70103

**Published:** 2025-05-08

**Authors:** Zoe Schindler, Elena Larysch, Felix Fornoff, Katja Kröner, Nora Obladen, Alexandra‐Maria Klein, Thomas Seifert, Christian Vonderach, Christopher Morhart

**Affiliations:** ^1^ Chair of Forest Growth and Dendroecology University of Freiburg Freiburg im Breisgau Germany; ^2^ Chair of Nature Conservation and Landscape Ecology University of Freiburg Freiburg im Breisgau Germany; ^3^ Department of Forest and Wood Science Stellenbosch University Stellenbosch South Africa; ^4^ Department of Biometry and Informatics Forest Research Institute Baden‐Württemberg Freiburg im Breisgau Germany

**Keywords:** bees, flowers, LiDAR, pollen, pollinator, remote sensing, terrestrial laser scanning, trees

## Abstract

Pollinator declines pose a threat to ecosystems and food production. Agriculture contributes to, but also suffers from, the erosion of pollination services. Our study explores the potential of trees in agricultural landscapes to support pollinators by providing floral resources. Our overarching objective is the quantification of floral resources produced by wild cherry (*Prunus avium* L.) that can be used by flower‐visiting and pollinating insects such as bees. Using an innovative approach, we combine pollen measurements with manual counts of flowers on branches and 3D data derived from terrestrial laser scanning. This approach allows us to scale up flower numbers from branches to entire trees. The derived models for estimating the probability of flower occurrence (*R* ^2^_c_ = 0.52, *R* ^2^_m_ = 0.50) and the number of flowers per branch (*R* ^2^_c_ = 0.88, *R* ^2^_m_ = 0.84), as well as the number of flowers per tree (*R* ^2^ = 0.83), show good model fits with only a small set of predictors. The model fits indicate that, at the branch level, predicting flowering probability is more challenging than predicting flower abundance. We found differences in the number of flowers per branch in different crown sections, suggesting that floral resources are heterogeneously distributed. Furthermore, we demonstrate that the number of flowers per tree increases exponentially with tree dimension (stem diameter, crown volume). Therefore, large trees provide disproportionately more floral resources than small trees and are particularly worthy of conservation efforts. For example, our models estimate that a single tree with a stem diameter of 25 cm carries 195,535 flowers (95% CI: 159,991–237,318), thus providing about 57 cm^3^ (95% CI: 32–88 cm^3^) of pollen and producing 170 g (95% CI: 48–345 g) nectar sugar per 24 h. This amount of pollen is sufficient to rear, for example, 5202 larvae (95% CI: 2886–8022) of *Lasioglossum laticeps*, a common and generalist sweat bee of cherry trees. In contrast, a smaller tree with a stem diameter of 10 cm provides only 8% of these resources. In conclusion, we demonstrate how our results contribute to the broader single‐large‐or‐several‐small debate in nature conservation by highlighting the value of large trees. Additionally, we show how information gathered at the branch level may be nondestructively upscaled to entire trees.

## INTRODUCTION

In the context of the ongoing biodiversity crisis, the abundance of terrestrial insects is estimated to be declining by around 10% per decade (van Klink et al., 2020). As part of this crisis, the abundance and diversity of pollinating insects are thought to be declining (Potts et al., 2010; Zattara & Aizen, 2021), which threatens the provisioning of pollination services (Klein et al., 2018; Potts et al., 2016). The security of the global food supply is highly dependent on the services provided by pollinating insects, as many crop species benefit to a certain degree from animal pollination (Klein et al., 2007). Declines in pollinator abundance and diversity can be attributed to various anthropogenic factors, including habitat loss, fragmentation, and degradation, as well as the use of agrochemicals and the impact of climate change (Potts et al., 2010; Rumohr et al., 2023; Wagner, 2020). Possible measures of action include mitigating climate change, reducing the use of pesticides (particularly insecticides), and protecting and enhancing habitats (Forister et al., 2019).

As the agricultural sector contributes to, but also suffers from, pollinator declines, this sector needs to be involved when seeking effective measures to mitigate these declines. An option to address most of these challenges is the inclusion of more flowering trees in agricultural systems. Agroforestry systems (AFS) are systems that combine livestock or crop production with woody perennials such as trees or shrubs, aiming for beneficial interactions between the components (Nair et al., 2021). By increasing habitat heterogeneity and connectivity, AFS are known to enhance biodiversity compared with conventional agriculture (Jose, 2009; Udawatta et al., 2019). Some of the groups benefiting from this are birds (Heath et al., 2017) and ground‐dwelling arthropods (Pardon et al., 2019), but also forest plants (Lenoir et al., 2021). AFS can also provide foraging resources for beneficial insects such as pollinators and may enhance their nesting resources (Bentrup et al., 2019; Kay et al., 2020), in turn promoting pollination services (Klein et al., 2003). Wild cherry (*Prunus avium* L.), for instance, provides large quantities of both nectar and pollen from March to April (Droege, 1984) and is assumed to improve habitat quality for pollinator insects (Kay et al., 2020). In addition, cherry trees are an attractive species for AFS because they are traditionally used for fruit production (Herzog, 1998) and can also be used to produce valuable high‐quality timber for the timber industry (Balandier & Dupraz, 1999; Coello et al., 2013).

To assess the beneficial effects of various tree and shrub species on pollinators, the floral resources of woody perennials in AFS, that is, nectar and pollen, must be quantified. However, quantifying flower abundance on trees and shrubs is time‐consuming and labor‐intensive. Further, the few studies estimating the number of flowers on individual trees have not focused on modeling floral abundance for trees of different dimensions or crown structures in detail. For example, Kay et al. (2020) examined coarse tree size classes and Carl et al. (2017) established that the number of flowers is correlated with tree dimensions. Apart from these two studies, we are not aware of any further studies assessing the number of flowers per tree and correlating these data with tree dimensions such as tree height, stem diameter, or crown area. Although the floral resources per tree vary with such tree dimensions (Carl et al., 2017; Kay et al., 2020) as well as with crown shapes and branch structures (Mika, 1992; Nasrabadi et al., 2022), models estimating the floral resources per tree based on tree dimensions, let alone models incorporating crown shape and branch structures, are still lacking. Especially in the context of AFS, where trees are frequently pruned or trained, predictive models that take tree dimensions, crown shape, and branch structures into account could improve estimates of floral resources provided by trees.

To acquire high‐resolution data on such structural information on trees, terrestrial laser scanning (TLS), a remote sensing technique based on Light Detection and Ranging (LiDAR), is currently the most promising technique providing highly accurate 3D position data in the magnitude of millimeters. From a multitude of individual laser distance measurements, accurate 3D representations of objects can be generated. This way, individual trees are represented by point clouds with millions of points (Dassot et al., 2011). To derive geometric, volumetric, and structural information from these point clouds, quantitative structure models (QSMs) can be employed. QSMs are simplified 3D models of trees that consist of geometric primitives such as cylinders and allow the derivation of single‐tree parameters such as trunk and branch size and volume as well as shape and size distribution (Disney et al., 2018). Further, the combination of TLS and QSMs is frequently used to obtain above‐ground tree volume or biomass (Calders et al., 2015; Disney et al., 2018; Gonzalez de Tanago et al., 2018; Schindler, Morhart, et al., 2023; Schindler, Seifert, et al., 2023) and is currently considered the most accurate nondestructive method for estimating these parameters.

The aim of this study was to establish models that predict the amount of floral resources provided by wild cherry trees based on tree size and crown structure. For this purpose, manual count data of the number of flowers of individual branches and 3D structures obtained using the TLS technique with QSMs were combined. These data were used to develop allometric models describing the number of flowers per branch, which were scaled up to estimate the number of flowers for whole trees. Finally, the pollen and nectar resources of all flowers per tree were summed, and the theoretical effects of the floral resources per tree on some exemplary bee species were estimated. Our specific research goals were as follows:

1. Modeling the number of flowers per branch based on branch and tree parameters, and testing the influence of branch position in the crown on the estimated number of flowers.
2. Upscaling the number of flowers from individual branches to entire trees by combining branch‐based regression models with TLS and QSMs.
3. Modeling the number of flowers per tree with statistical models based on simple tree dimensional parameters and estimating the available nectar and pollen per tree and the number of bees that can be sustained using these resources.

On top of these research objectives, we addressed the following three research questions:

1. Light availability is not evenly distributed within tree crowns, with typically more radiation reaching the top of the crowns. Furthermore, insect pollination relies not only on olfactory but also on visual cues (Barragán‐Fonseca et al., 2020; Rachersberger et al., 2019). Thus, high visibility of flowers in outer crown parts might be advantageous for pollination success. For these reasons, we asked: does branch position within the crown influence flower abundance?
2. Most allometric functions for trees, for example, tree volume and biomass, are nonlinear (Picard et al., 2012). Therefore, we asked: is the relationship between tree or crown dimension and flower abundance at the tree level nonlinear as well?
3. Tree related allometric functions typically use the dbh as a predictor. However, since the flowers are located within the crown, we expect crown‐related parameters to better capture the relationship between tree dimension and floral abundance. Thus, we asked: do crown‐related variables capture flower abundance better than dbh?

## MATERIALS AND METHODS

Our multilevel method is based on manual flower counts on branches in different crown sections and on 3D tree models constructed using TLS and QSMs. By combining these data sets, the number of flowers is scaled up from the branch to the tree level. Additional field data on the pollen content of flowers is used to estimate the forage resources for pollinators of trees with different dimensions. In the following, the data collection and analysis of the manual count data, the remote sensing data, and the pollen data are described in more detail.

### Study sites

**FIGURE 1:**
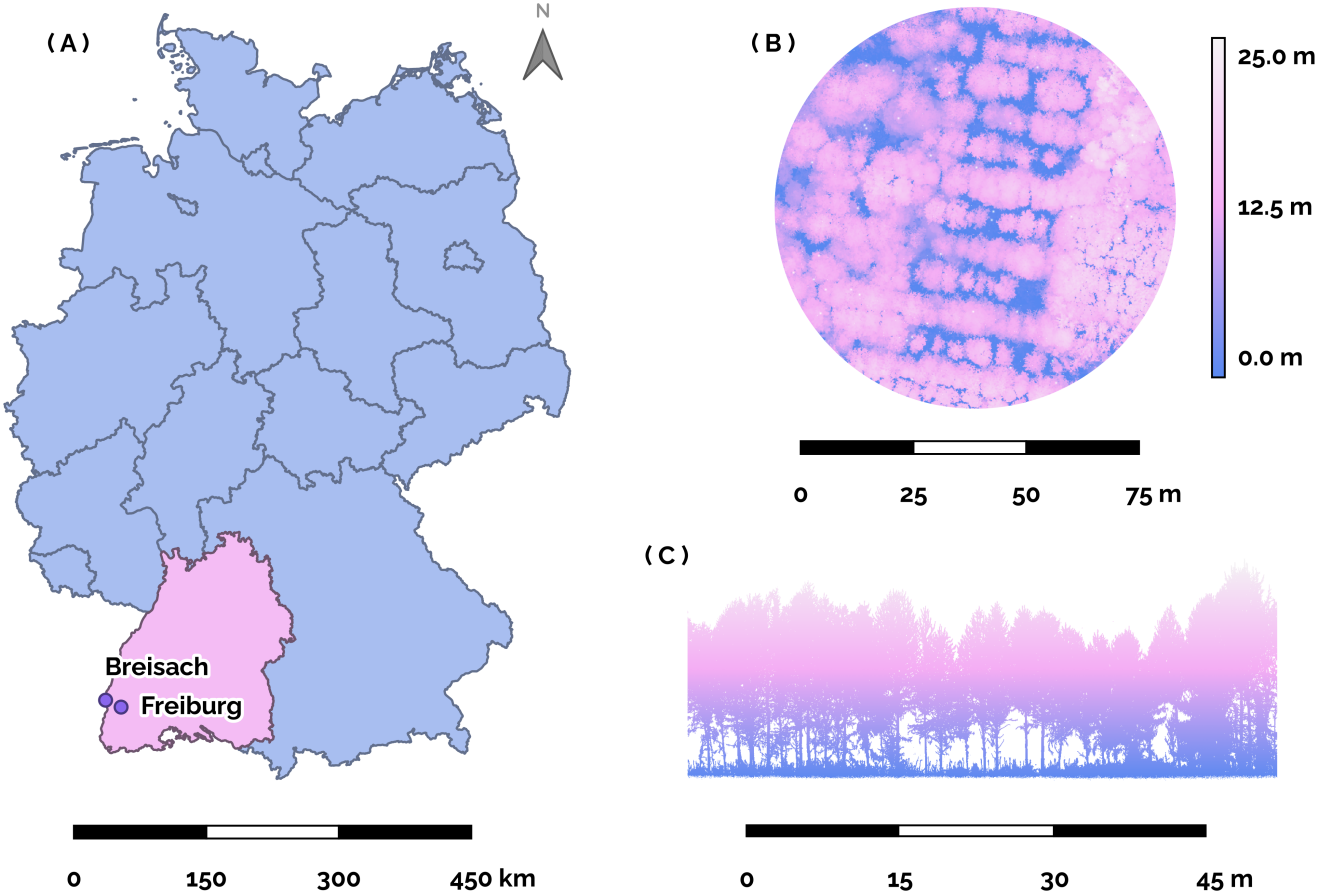
Location and overview of the study site. (A) Map of Germany (blue and pink area), the state of Baden‐Württemberg (pink area), and the cities of Freiburg and Breisach (purple points), Geodata: ©GeoBasis‐DE/BKG (2023). (B) Canopy height model (top view) of the study site (colors indicate the height above‐ground). (C) Cross section of the point cloud for one of the tree rows.

The sampled trees for the manual flower count data and for the 3D data collected using TLS were located on a study site close to the city of Breisach (48°4′13″ N, 7°35′23″ E, 188 m above sea level) in Southwest Germany (Figure 1). The data on the pollen content of cherry flowers was collected from wild cherry trees at several locations around Freiburg and Breisach to cover a larger diversity of varieties (see *Estimating foraging resources for bees*).

The study site was a plantation of wild cherry (*Prunus avium* L.), pedunculate oak (*Quercus robur* L.), common ash (*Fraxinus excelsior* L.), sycamore (*Acer pseudoplatanus* L.), small‐leaved linden (*Tilia cordata* Mill.), and common hornbeam (*Carpinus betulus* L.). The site currently has a total size of 2.5 ha and was established in 1997 with the primary objective of studying the production of valuable wood in a widely spaced plantation akin to an AFS. Since plantation establishment, selected trees with straight stems have been pruned with the aim of producing high‐value timber. The soil was formed from sediments of the Rhine with high water permeability and low water storage capacity. The site is therefore prone to drought in dry and hot summers. The closest weather station of the German Weather Service (Deutscher Wetterdienst) is situated in the city of Freiburg (Figure 1). In the climatic period from 1991 to 2020, the long‐term average air temperature and precipitation sum amounted to 11°C and 896 mm, respectively. During the vegetation period, from April to September, the long‐term average air temperature and precipitation sum were 16.5°C and 510 mm, respectively (Deutscher Wetterdienst, 2023).

### Manual count data

**FIGURE 2:**
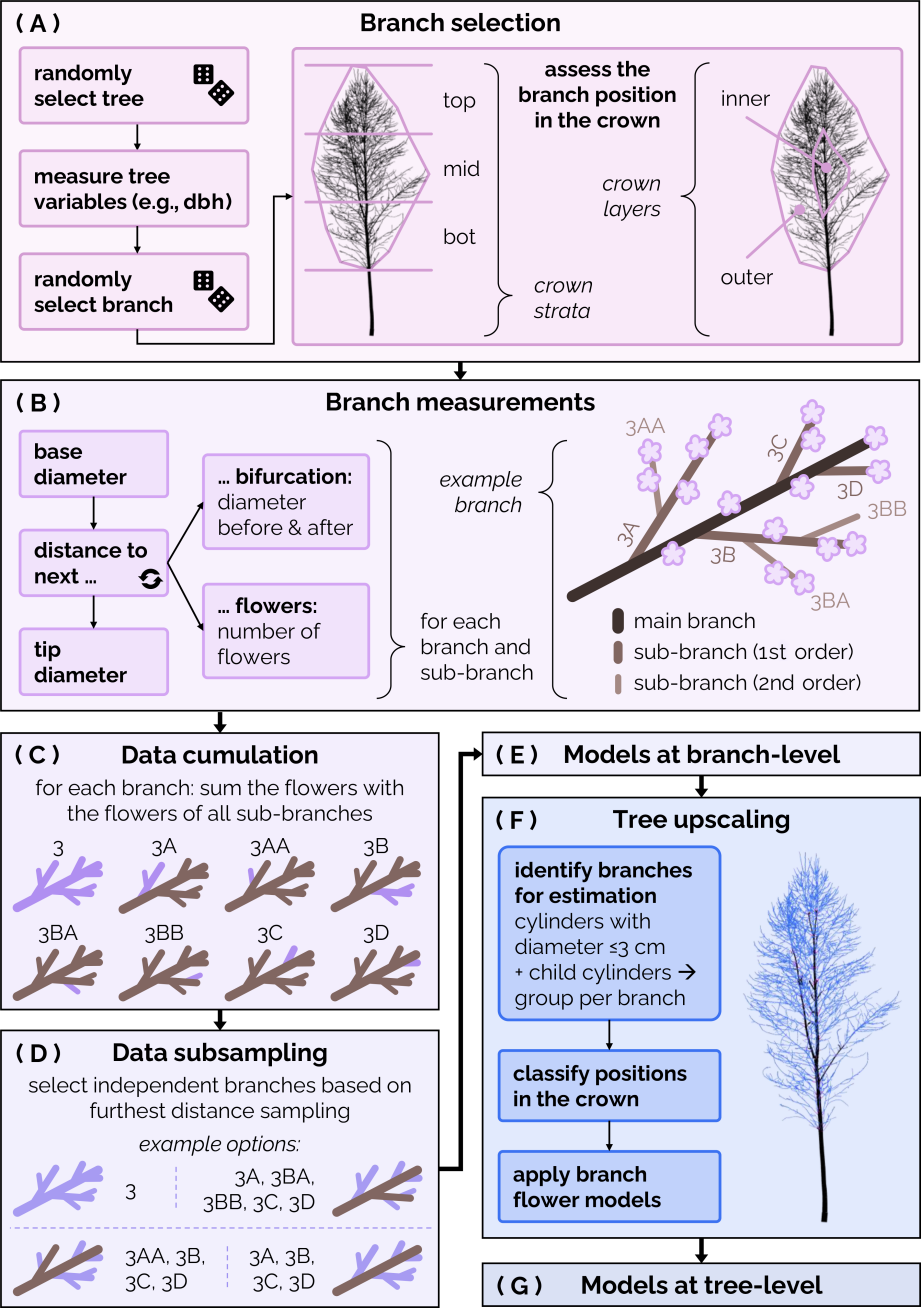
Visualization of the workflow from data collection to tree‐level models. (A) Tree and branch selection including the crown stratification into three vertical crown strata and inner and outer layers. (B) Branch measurements and nomenclature of branches and subbranches. (C) Data cumulation of each branch with its respective subbranches. (D) Data subsampling to obtain a subset of independent data points. (E) Modeling the number of flowers per branch. (F) Upscaling the number of flowers per branch to the number of flowers per tree using 3D tree models. (G) Modeling the number of flowers per tree. Credit: Zoe Schindler.

To assess flower abundance and distribution along branches and twigs, a total of 66 branches belonging to different branch orders were cut off from 16 wild cherry individuals (see Figure 2A). The branches were chosen randomly, but we attempted to sample different crown sections from each tree to acquire representative data. The trees were randomly selected from the research site, provided they appeared healthy and were flowering at the time of data collection. The trees had a stem dbh (measured at 1.3 m above the ground) between 9 and 31 cm (mean 20 cm; SD 6 cm) and were sampled manually in spring 2022 and 2023. When the branches and all their subbranches are counted as individual branches, 827 branches with a cut‐off diameter of 2–55 mm were sampled. As not all branches were cut directly at the branch base, “branch diameter” in the following refers to the branch diameter where the branches were cut off, which does not always coincide with the branch base diameter.

The position of all branches in the crowns was recorded by visually dividing the crown into different sections (see Figure 2A). This stratification of the crown was performed to roughly estimate the light availability within the crown. Starting at the crown base height, the crown of each tree was stratified into three vertical strata of the same relative length (1/3 of the crown length): the bottom (bot), middle (mid), and top (top) crown. In addition, the crown was stratified into an outer (out) and inner (in) layer. The outer layer was defined as the 2 m from the crown surface inward, while the inner layer included the remaining crown parts. Additionally, the crown was divided into eight compass directions (N, NE, E, SE, S, SW, W, NW). While in the case of small branches, an attempt was made to select only branches that were clearly located in one of the crown sections, it was sometimes difficult to assign larger branches exclusively to one of these sections. Therefore, large branches whose parts were located in several crown sections were assigned to the crown section in which the largest part of the length of the main shoot was located.

After branch removal, measurements were taken according to Figure 2B. For each branch, first the main shoot and then all its subbranches were measured and flowers counted. The measurement procedure consisted of a series of length and diameter measurements along the branch axis as well as flower counting. The detailed measurements allowed for the virtual division of the branches into branch diameter classes in 5‐mm intervals (i.e., 0–5 mm, 5–10 mm). As the diameter classes sometimes changed between diameter measurements, the exact shoot length in each diameter class was calculated assuming a linear diameter change between two consecutive diameter measurements (see Appendix S1: Figure S1). To increase the number of thick branches in the data set more efficiently, additional larger branches were randomly sampled for which only the initial branch diameter and the total number of flowers were recorded (24 of 66 branches). Sampling large branches with the detailed measurements would not have been feasible. These branches were sampled additionally to improve the robustness of the model predictions for larger diameters, allowing the model to be used exclusively within the interpolation range for the subsequent upscaling. The data sets from both the detailed and the simplified measurements were later used for modeling the number of flowers per branch. The precisely measured branches were also used to determine the diameter classes to which the flowers were directly attached. This information was essential for the subsequent upscaling to entire trees.

Since our aim was to determine the total number of flowers on each branch, including all flowers on the respective subbranches, the number of flowers of each branch was summed with the flowers of all corresponding subbranches (see Figure 2C; Appendix S1: Figure S2).

### Remote sensing data

In total, 39 wild cherry trees with a dbh from 9 to 28 cm (mean 18 cm, SD 5 cm) were measured using TLS on the same site where the branch measurements were taken. The scanning using a RIEGL VZ‐400i (RIEGL Laser Measurement Systems GmbH, Horn, Austria) was conducted in two campaigns during winter dormancy in March 2022 and in January 2023. The trees were surveyed using a multi‐scan approach, with at least four scans per tree from multiple directions. The scanner was set to a pulse repetition rate of 1200 kHz and an angular resolution of 0.04° vertically and horizontally.

After data acquisition, the data were preprocessed using the software RiSCAN PRO v2.16.3 (RIEGL Laser Measurement Systems GmbH, Horn, Austria). The pre‐processing steps included co‐registering, filtering, merging, and down‐sampling of the point clouds. Co‐registration describes the process of aligning individual point clouds from different scanning positions with each other. The co‐registration was done automatically based on the recorded scanner position and the point clouds themselves. All point clouds were filtered for isolated points (<5 points in 10 cm neighborhood), dark points (reflectance ≤−15 dB), scattered points (deviation ≥15), and points recorded at a large distance to the scanner (range ≥50 m). To reduce co‐registration errors, a multi‐station adjustment (MSA2) was applied on the filtered point clouds (Demol et al., 2022). Finally, the point clouds were merged and down‐sampled to 1 point/cm^3^ to homogenize point density and increase computational efficiency of subsequent steps. Next, the point cloud was segmented into single‐tree point clouds using CloudCompare v2.11.3 (CloudCompare, 2022). Remaining noise was removed manually during single‐tree segmentation.

The single‐tree point clouds were used to reconstruct QSMs, simplified 3D tree models based on geometric primitives, using TreeQSM v2.4.1 (Raumonen & Åkerblom, 2022) in MATLAB R2023a (The MathWorks Inc., Natick, Massachusetts, USA). As the QSM reconstruction process depends upon a set of hyperparameters, a hyperparameter optimization was conducted. Different sets of hyperparameters were tested dependent on tree height, comprising 12 hyperparameter sets per tree. As the TreeQSM reconstruction process is based on stochastic elements, multiple QSMs had to be calculated to obtain stable estimates. Therefore, for each tree and set of hyperparameters, five QSMs were fitted. Using the optimal combination of hyperparameters for each tree, as indicated by the lowest average distance between points and fitted cylinders of the five QSMs of one hyperparameter combination, a final set of 30 QSMs per tree was derived. According to the TreeQSM manual, five models are sufficient to robustly (with an error of only a few percent) estimate the average of variables (Raumonen & Åkerblom, 2022). We chose to reconstruct more QSMs to further increase the precision of the mean estimates. For all QSMs, the minimum cylinder diameter was decreased from the default value of 5 to 3 mm. This allows the software to fit smaller cylinders that better reflect the observed branch diameters.

From the QSMs, various tree dimension parameters were derived: dbh, tree height, crown diameter, crown projection area, and crown volume. These variables were directly obtained from the TreeQSM output, and their calculation is described in more detail in the TreeQSM manual (Raumonen & Åkerblom, 2022). Crown projection area describes the area that is covered by the vertical projection of the tree crown onto the ground, delineated by a convex hull. Crown volume is obtained from a 3D alpha shape fitted around all of the cylinders belonging to the crown. All subsequent data analyses were conducted in the programming language R v4.2.2 (R Core Team, 2023). All raw data and code were made available at Schindler et al. (2025).

### Statistical analysis

The manually collected data were used to fit a regression model relating branch (branch ID, diameter, inner/outer crown layer, bottom/middle/top crown stratum, compass direction) and tree variables (tree ID, dbh) to the number of flowers per branch. To avoid pseudo‐replication, a sample of branches was drawn from all branches and subbranches (see Figure 2D). We opted for a subsampling method, as the inclusion of complex branching hierarchies with different branching depths is not feasible with simple nested random effects. The subsampling was based on a farthest‐distance sampling approach to achieve a balanced data set. In this approach, every branch is represented by a point in an *n*‐dimensional space, with the *n* dimensions being the number of variables considered. Here, we used six parameters: branch diameter, crown layer, crown stratum, compass direction, tree ID, and dbh. These variables are converted to numerical values and are scaled to ensure similar importance. Starting with the branch with the largest diameter, the Euclidean distance toward all other branches across the *n*‐dimensional space is calculated. Then, the branch which is located farthest away, that is, which is the most different, is selected as the next branch. Once a branch is selected, all its subbranches and parent branches are removed from the further selection process to obtain independent data points which contain no information of other data points and whose information is not included indirectly in other data points. This subsampling reduced the data from 827 branches, which is the total number of measured branches and subbranches, to 308 branches.

The subsampled data was then used to fit models for estimating the number of flowers per branch (see Figure 2E). To intercept any remaining similarities between subbranches extracted from the same cut‐off branch, each cut‐off was assigned a unique “branch ID”. The branch ID was used as a factor when modeling the intercept by including a random effect. To enable the use of random effects, mixed‐effects models were employed. To overcome zero‐inflation and overdispersion, we chose a two‐part hurdle model consisting of a binomial and a zero‐truncated negative binomial model. The binomial regression was used to model whether a flower occurs on a branch or not (Equation 1), while the zero‐truncated negative binomial regression estimates the flower abundance based on the results of the first regression (Equation 2). Model selection was done separately for each model by manual stepwise reduction of the full model, comparison of the respective models using likelihood ratio tests (with *p* < 0.05) and checking the residuals of the derived models for patterns. If there was no statistical difference between models, the simpler model was chosen. The full models contained the dbh, the logarithm of the branch diameter, the crown strata, the crown layers, and the compass directions, as well as interactions between the dbh and the logarithm of the branch diameters and between crown strata and crown layers.

(1) $$\text{logit}\left({\mathrm{FP}}_{b}\right)=\beta_{0}+\beta_{1}\times \ln\left(D\right)+\beta_{2}\times \mathrm{dbh}+\beta_{3}\times C_{l}+\beta_{4}\times C_{s}+\beta_{5}\times C_{l}\times C_{s}+b_{\mathrm{bt}}+\varepsilon$$

(2) $$\ln\left({\mathrm{FN}}_{b}\right)=\beta_{0}+\beta_{1}\times \ln\;\left(D\right)+\beta_{2}\times \mathrm{dbh}+\beta_{3}\times C_{l}+\beta_{4}\times C_{s}+\beta_{5}\times \ln\;\left(D\right)\times \mathrm{dbh}+\beta_{6}\times C_{l}\times C_{s}+b_{\mathrm{bt}}+\varepsilon$$

The final model equations of the two model parts are given in Equations (1) and (2), where ${\mathrm{FP}}_{b}$ is the probability of flower occurrence and ${\mathrm{FN}}_{b}$ is the number of flowers on a branch. The variables $\beta_{0}$ to $\beta_{6}$ are model coefficients. D is the branch diameter (in millimeters) and dbh is the dbh (in centimeters). $C_{l}$ denotes the crown layers (in, out), while $C_{s}$ denotes the crown strata (bot, mid, top). In both models, $C_{l}$ and $C_{s}$ were implemented as factors. The random intercept for the branch ID of the cut‐off branch is nested in a random intercept for the respective tree ID and is described by $b_{\mathrm{bt}}$. The residual error is given as ε. In both model parts, the variables describing crown layers and crown strata remained in the model after model selection, while compass direction was not included.

To answer our first question regarding the influence of branch position within the crown on floral abundance, we estimated marginal means for both model parts using the emmeans package (Lenth, 2023). Subsequently, pairwise comparisons on the different levels of the crown variables were conducted on these marginal means, with *p*‐values adjusted using Tukey's procedure.

In the next step, the branch‐level models were upscaled to entire trees using QSMs (see Figure 2F). To apply the models to the QSMs, the crowns of the tree models had to be deconstructed into individual branches to which the branch‐level models could be applied. For this, all QSM cylinders with a diameter ≤3 cm were identified. For each of these cylinders, the consecutive cylinders of the branch and the subbranches were selected. Duplicates, resulting from a cylinder being selected individually and as part of another branch, were removed. The result of this step was a collection of branch tips (including subbranches) starting at a maximum diameter of 3 cm, as well as whole branches (including subbranches) with a branch base diameter ≤3 cm that were attached to cylinders of parent branches of diameter >3 cm. The applied threshold of 3 cm is a fair compromise between the reliable diameter range of the model and the QSMs (see Appendix S1: Figure S2). While the branch‐level model was fit primarily using data from branches with small diameters, QSMs are known to become less precise with decreasing branch diameters (Demol et al., 2022; Morhart et al., 2024). As flowers were mainly attached to branches and spurs with a diameter <2 cm (see 2.3), using a threshold of 3 cm ensures that all branch parts potentially bearing flowers were used for the upscaling. By refraining from applying the models to thinner branches, we reduce the potential impact of QSM uncertainty at very small branch diameters.

For each of the identified branches and branch tips, their position in the crown had to be estimated according to the stratification of the crown (Figure 2A). For this purpose, a bounding box was fitted for each identified branch part (including its subbranches), extending from the minimum to the maximum *XYZ* coordinates. The center of the box was then classified into the three crown strata. For the classification into the inner and outer part of the crown, the entire crown was virtually “sliced” into a stack of cross sections, each measuring 0.5 m in height (see Appendix S1: Figure S3). Each of these cross sections was projected onto the *xy*‐plane, and a convex hull around the points was calculated. The outline of the hull was then buffered by 2 m. Points within this buffer were assigned to the outer crown, while points to the interior of this buffer were assigned to the inner crown. After the classification into the six crown sections, the binomial model (Equation 1) was used to predict the probabilities of flower occurrence. Using these probabilities, it was randomly determined for each branch whether it carried flowers. For those predicted to bear flowers, the number of flowers was determined using the second part of the model (Equation 2). Finally, the predicted number of flowers per branch was summed for each QSM. As there were multiple QSMs per tree, the median number of flowers for each tree across all models was calculated and used in further steps.

Based on these estimates, regressions were derived to estimate the number of flowers per tree (see Figure 2G), using tree dimensions (dbh, height) and crown dimensions (diameter, projected area, volume) as predictors. For this purpose, Pearson's correlation coefficient (*r*) was calculated for the number of flowers and tree dimension parameters such as dbh (*r* = 0.86), tree height (*r* = 0.42), crown diameter (*r* = 0.73), crown projection area (*r* = 0.72), and crown volume (*r* = 0.82). Since all correlations between flower abundance and tree dimensions were significant (*p* < 0.05) but correlations with dbh and crown volume were the strongest, these two parameters were selected to subsequently fit models estimating the number of flowers per tree based on tree and crown dimensions. Since dbh and crown volume were strongly correlated with each other (*r* = 0.82, *p* < 0.001), indicating collinearity (|*r*| > 0.7, Dormann et al., 2013), two separate models using these variables were fitted in the next step.

Our second question about the nonlinearity of the relationship between tree dimension and floral abundance was addressed by fitting linear regressions between dbh and floral abundance (Equation 3), as well as between crown volume and floral abundance (Equation 4). Since the residuals of the simple linear regressions were not normally distributed (Kolmogorov–Smirnov tests, *p* < 0.001), indicating a nonlinear relationship between the variables, a negative binomial distribution with the natural logarithm as a link function was used. We decided against forcing the intercept of the model through the origin, as we assumed that very small and young trees likely behave differently in their flowering behavior anyhow, as trees usually only reproduce starting at a certain age. The model equations for the tree‐level models are given in Equations (3) and (4), where ${\mathrm{FN}}_{t}$ is the number of flowers on a tree, $\beta_{0}$ and $\beta_{1}$ are the model coefficients, dbh is the dbh (in centimeters), CV is the crown volume (in cubic meters), and ε is the residual error. No random effect was used in these models as we used the median of the dbh, crown volume and number of flowers of the 30 QSMs per tree. Thus, we were left with only one data point per tree (*n* = 39).

(3) $$\ln\left({\mathrm{FN}}_{t}\right)=\beta_{0}+\beta_{1}\times \mathrm{dbh}+\varepsilon$$

(4) $$\ln\left({\mathrm{FN}}_{t}\right)=\beta_{0}+\beta_{1}\times \mathrm{CV}+\varepsilon$$

To address our third question about the relationship between dbh, crown variables, and flower abundance per tree, we compared the performance metrics (*R* ^2^, RMSE, MAE) of the models using dbh or crown volume as predictors, respectively.

All models were fitted using the glmmTMB package (Brooks et al., 2017). Model diagnostics were derived using the DHARMa package (Hartig, 2022) and are listed in Appendix S1. For each model, basic checks on the uniformity of the residuals, dispersion, and the occurrence of outliers were conducted (see Appendix S1). The models were assessed with the respective data set using the marginal (m) and conditional (c) *R* ^2^ (*R* ^2^_m_, *R* ^2^_c_), mean absolute error (MAE_m_, MAE_c_) and root mean square error (RMSE_m_, RMSE_c_). Marginal statistics were calculated using only fixed effects for predictions, while conditional statistics were calculated incorporating random intercepts. *R* ^2^ values were calculated using the MuMIn package (Bartoń, 2023). Further, models were validated using a leave‐one‐out cross‐validation not incorporating random intercepts (MAE_cv_, RMSE_cv_).

### Estimating foraging resources for bees

As a basis for modeling the floral resources, pollen volume and nectar production per 24 h were used. The number of pollen grains per flower and pollen diameters were assessed for six different trees. For pollen quantification, short branches with open and unopened flowers were collected and stored for one night indoors. Following Rowe et al. (2020), from each tree, the stamens from 10 freshly opened flowers with fully ripened stamens were extracted, preserved in alcohol, and filtered to remove non‐pollen material. Then, the solution was centrifuged, mixed with water, and the number of pollen grains in a subsample of the solution was counted using a microscope and a chamber slide with a fixed volume. On average, one flower contained 16,059 pollen grains (SE 3327; SD 8151). Finally, the diameters of three pollen grains per tree were measured and averaged. Using the average pollen diameter over all sampled trees, the average pollen volume was estimated using the volume of a sphere. The diameter of the sampled pollen grains averaged 32.63 μm (SE 0.73 μm; SD 1.78 μm). The average pollen volume was multiplied by the average pollen count per flower to estimate the total pollen volume per flower. According to this calculation, one flower contained on average 0.29 mm^3^ of pollen. For nectar and sugar quantification, we assumed a nectar production of 3.7 mg/flower/24 h with a sugar content of 23.5%, based on Droege (1984), who provided a range for both nectar production (1.4–6.0 mg/flower/24 h) and sugar content (10%–37%) for *Prunus* spp. For our calculations, we used the arithmetic mean of the lower and upper limits. The daily nectar and sugar production per tree were calculated by multiplying the number of flowers per tree by the nectar production of a single flower.

Using data and models from Müller et al. (2006) and Westrich (2019), the number of bee larvae that can be reared using the floral resources of differently sized trees was estimated (see Appendix S1: Section S1). As a multitude of bee species forages on wild cherry and since pollen requirements vary with bee size, we calculated the pollen requirements for three differently sized wild bee species that commonly visit wild cherry (*Andrena helvola* L.; *Bombus terrestris* L.; *Lasioglossum laticeps* Schenk) and for the commercially used honeybee (*Apis mellifera* L.).

The 95% CIs for pollen content, daily nectar sugar production, and the number of supported bee larvae were derived using a combination of bootstrapping and Monte Carlo simulations to incorporate the uncertainty from the number of pollen grains per flower, pollen diameter, nectar production, and nectar sugar content. In each of the 1000 repetitions of the bootstrap on the model relating the number of flowers per tree to dbh, pollen volume and daily nectar sugar production were estimated using randomly drawn values for the number of pollen grains per flower, pollen diameter, nectar production, and nectar sugar content. We assumed a normal distribution for these four parameters and used the mean of the measured values or provided ranges as the mean in the respective parameter distributions. For the number of pollen grains per flower and pollen diameter, we calculated the SE of the mean and used it as the SD in the parameter distributions. In the case of the ranges for nectar production and sugar content, we assumed that the given range covers the 95% CIs, that is, the mean ± 2 × SE. Therefore, the range differences were divided by four and then used as SDs in the parameter distributions. Based on the predicted pollen volume, the number of supported larvae was estimated using the previously determined pollen requirements per bee larva. From the 1000 predictions per dbh value, the 2.5% and 97.5% quantiles were calculated to derive the 95% CIs.

## RESULTS

**FIGURE 3:**
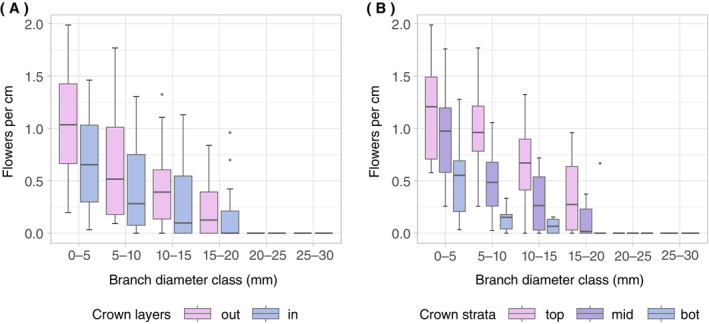
Flowers per centimeter branch within different branch diameter classes. (A) Number of flowers per branch centimeter divided into the inner (in) and outer (out) crown layers. (B) Number of flowers per branch centimeter divided into crown strata (top, mid, bot). The error bars show the minimum and maximum values in the data that were not below the 25% quantile or above the 75% quantile ±1.5 times the inter‐quantile range. The outlier points indicate values outside this range.

The selected 308 branches of the initial 827 branches ranged in their cut‐off diameter from 3 to 55 mm (mean 9 mm; SD 6 mm). Of these branches, 13% did not produce any flowers. The remaining branches carried between one and 2073 flowers (mean 107; SD 259). The segmentation of branches into diameter classes revealed that flowers were exclusively on branch segments and spurs with diameters of less than 2 cm. Visual inspection suggests that flower density in the diameter classes decreased with increasing branch diameter and varied with the position in the crown, with higher flower density in outer and upper crown parts (Figure 3).

**FIGURE 4:**
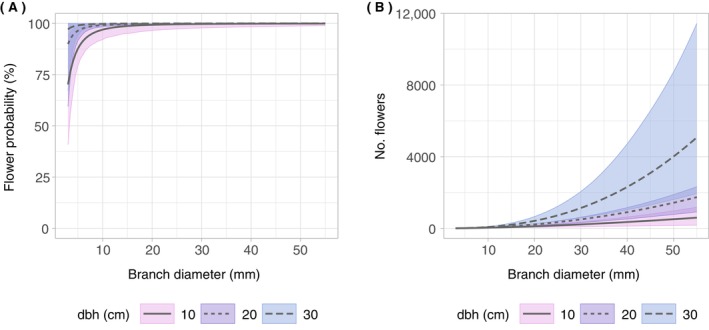
Effect plots of the two‐step branch‐level model relating the number of flowers to branch and tree parameters. To isolate the effects of branch diameter and dbh, the categorical predictors were set to the most frequent classes (crown stratum: bot, crown layer: out). The shaded areas correspond to the bootstrapped (*k* = 1000) 95% CIs. (A) Flower probability as a function of branch diameter (in millimeters) and dbh (in centimeters). (B) Number of flowers as a function of branch diameter (in millimeters) and dbh (in centimeters).

**FIGURE 5:**
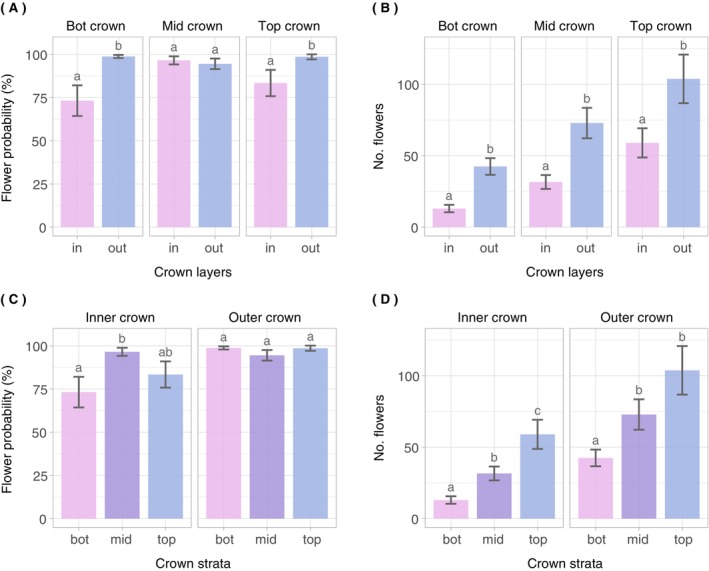
Estimated marginal means derived from the fitted branch‐level models. Significant differences (*p* < 0.05) are indicated with letters. The error bars indicate the SEs of the estimated means. (A) Flower probability depending on crown layer. (B) Number of flowers depending on crown layer with an interaction with crown stratum. (C) Flower probability depending on crown stratum. (D) Number of flowers depending on crown stratum with an interaction with crown layer.

While the model for predicting the probability of flower occurrence on branches showed a moderate fit (Table 1, *R* ^2^_c_ = 0.52), the model for the number of flowers on branches showed a good fit (Table 1, *R* ^2^_c_ = 0.88). With increasing branch diameter and dbh, both the probability of flower occurrence and the number of flowers increased (Table 2, Figure 4). The probability of flower occurrence approached 100% at branch diameters above 2 cm (Figure 4A). While the number of flowers per branch increased with branch diameter, the nonlinearity of this relationship only became apparent at larger dbh values (Figure 4B). This model behavior can be explained by the interaction between dbh and branch diameter in the model formula (Equation 2). With regard to the branch positions in the crown, according to estimated marginal means, both the probability of flower occurrence and the abundance of flowers were significantly different (*p* < 0.05) among the crown sections (Figure 5, Appendix S1: Table S5). Generally speaking, flower probability and abundance increased from bot < mid < top (crown strata) and from in < out (crown layers). Overall, the effects of branch diameter, dbh, and crown position were more pronounced and unequivocal for the number of flowers than for the probability of flower occurrence (Figures 4 and 5).

**TABLE 1:** Model fits of the branch‐ and tree‐level models.

Model	*R* ^2^_c_	*R* ^2^_m_	MAE_c_	MAE_m_	MAE_cv_	RMSE_c_	RMSE_m_	RMSE_cv_
Branch								
Flower occurrence	0.52	0.50	0.16	0.17	0.18	0.28	0.29	0.31
Flower abundance	0.88	0.84	35	67	56	99	232	176
Combined model	n.a.	n.a.	33	61	51	93	217	166
Tree								
Flower abundance ~ dbh	n.a.	0.83	n.a.	20,357	21,800	n.a.	31,291	34,334
Flower abundance ~ crown volume	n.a.	0.68	n.a.	25,858	27,691	n.a.	37,011	39,557

*Note*: The branch‐level model is a two‐part hurdle model predicting flower occurrence (Equation 1) and abundance (Equation 2) separately. *R* ^2^_c_, MAE_c_, and RMSE_c_ describe the predictive capabilities on the training data when incorporating the random effect. *R* ^2^_m_, MAE_m_, and RMSE_m_ describe the predictive capabilities on the training data when not incorporating the random effect. MAE_cv_ and RMSE_cv_ describe the predictive capabilities on test data as derived by leave‐one‐out cross‐validation.

**TABLE 2:** Model description of the two‐part branch‐level model relating flower count to branch and tree parameters.

Model specification	Predictor	Unit	Estimate	SE
Flower occurrence ~ ln(diameter) + dbh + crown layers × crown strata + (1|tree/branch)	(Intercept)		−2.85	1.25
	ln(diameter)	mm	2.16	0.67
	dbh	cm	0.13	0.06
	Crown layers (inner)		−3.38	0.74
	Crown strata (middle)		−1.54	0.77
	Crown strata (top)		−0.15	1.24
	Crown layers (inner) × crown strata (middle)		3.86	1.09
	Crown layers (inner) × crown strata (top)		0.76	1.34
Flower abundance ~ ln(diameter) × dbh + crown layers × crown strata + (1|tree/branch)	(Intercept)		0.42	0.71
	ln(diameter)	mm	1.22	0.34
	dbh	cm	−0.06	0.04
	Crown layers (inner)		−1.18	0.23
	Crown strata (middle)		0.54	0.18
	Crown strata (top)		0.89	0.22
	ln(diameter) × dbh		0.04	0.02
	Crown layers (inner) × crown strata (middle)		0.35	0.29
	Crown layers (inner) × crown strata (top)		0.62	0.35

*Note*: The first model part describes the probability of flower occurrence using the binomial distribution and the logit link function (Equation 1). The second model part describes the flower abundance upon flower occurrence using a zero‐truncated negative binomial distribution and the natural logarithm as the link function (Equation 2). The table gives the model specifications (in R syntax) as well as the estimated effects and their SEs.

In total, 39 trees within a dbh range of 9 to 28 cm (mean 18 cm; SD 5 cm) and a height of 7.9 to 20.1 m (mean 15.6 m; SD 2.4 m) were remotely sensed. Crown diameter ranged from 3.1 to 7.3 m (mean 5.2 m; SD 1.1 m), crown projection area ranged from 9.6 to 55.5 m^2^ (mean 28.0 m^2^; SD 11.7 m^2^), and crown volume ranged from 13.8 to 282.7 m^3^ (mean 121.4 m^3^; SD 71.8 m^3^). Based on our upscaling approach, we estimated these trees to carry 6540 to 246,245 flowers (mean 76,898; SD 68,056).

**FIGURE 6:**
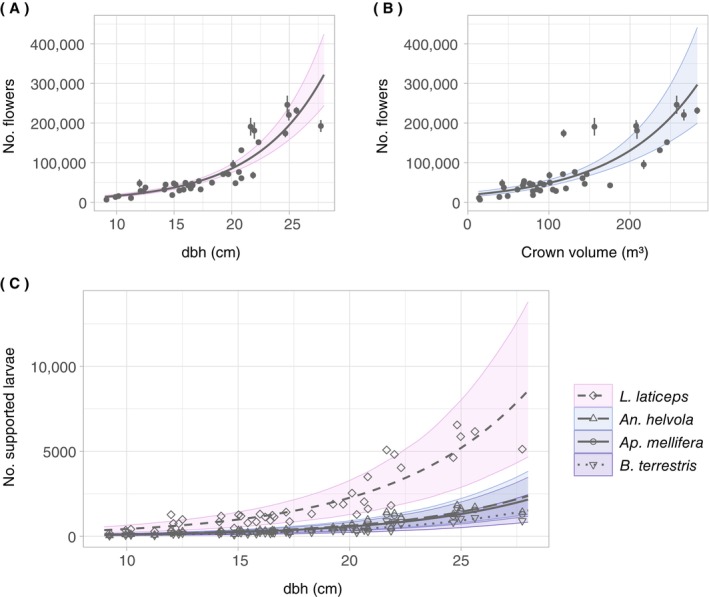
Effect plots of the tree‐level models relating the number of flowers to tree parameters. (A) Number of flowers as a function of dbh (in centimeters). (B) Number of flowers as a function of crown volume (in cubic meters). (C) Number of supported larvae for selected bee species (*Andrena helvola* L.; *Bombus terrestris* L.; *Lasioglossum laticeps* Schenk; *Apis mellifera* L.) as a function of dbh (in centimeters). The error bars in (A) and (B) show the uncertainty of the QSM (quantitative structure model) reconstruction with median ± SD. In all subplots, the shaded areas show the 95% CIs of the models. For the CIs in (C), the model for estimating the number of flowers per tree based on dbh was bootstrapped 1000 times, and Monte Carlo simulations were used to incorporate the uncertainty from the estimates of the number of pollen grains per flower and the pollen diameter.

The model relating the number of flowers per tree to dbh (Equation 3, *R* ^2^ = 0.83) outperformed the model based on crown volume (Equation 4, *R* ^2^ = 0.68) in terms of *R* ^2^, RMSE, and MAE (Table 1). In both models, the respective predictor showed highly significant (*p* < 0.001) effects on the number of flowers per tree (Table 3). Moreover, both models showed a nonlinear relationship between the number of flowers and the respective predictor (Figure 6A,B). Thus, trees with a larger dbh or crown volume were predicted to carry considerably more flowers and associated resources than smaller trees. For example, the model predicts that a tree of dbh 10 cm will bear 16,163 flowers (95% CI: 12,311–20,723). The associated pollen volume and daily nectar sugar production are estimated at 5 cm^3^ (95% CI: 3–7 cm^3^) and 14 g (95% CI: 4–29 g), respectively (see Appendix S1: Figure S8). In contrast, a tree with a dbh of 25 cm is estimated to bear approximately 195,535 flowers (95% CI: 159,991–237,318), to provide 57 cm^3^ (95% CI: 32–88 cm^3^) of pollen, and to produce 170 g (95% CI: 48–345 g) nectar sugar per 24 h. This corresponds to an increase in dbh by a factor of 2.5, but an increase in the number of flowers and associated resources by a factor of 12.

**TABLE 3:** Model descriptions of the two models relating the logarithm of the number of flowers per tree to dbh (Equation 3) and crown volume (Equation 4).

Model	Predictor	Unit	Estimate	SE	*p*‐value
Tree flower abundance ~ dbh	(Intercept)		8.03	0.22	<0.001
	dbh	cm	0.17	0.01	<0.001
Tree flower abundance ~ crown volume	(Intercept)		9.80	0.16	<0.001
	Crown volume	m^3^	0.01	0.00	<0.001

*Note*: Both models use the negative binomial distribution and the natural logarithm as the link function. The table gives the model specifications as well as the estimated effects, SEs, and *p*‐values.

The nonlinear relationship between dbh and the number of flowers per tree is also evident in the estimated number of supported bee larvae (Figure 6C), as the pollen volume per tree was linearly related to the number of flowers per tree. Among the selected bee species, this was most apparent in the sweat bee species *L*. *laticeps*, whose individuals are considerably smaller than those of the other selected species and therefore require considerably less pollen per larva. While a tree with a dbh of 10 cm could provide pollen for 77 larvae (95% CI: 43–116) of *B*. *terrestris* or 430 larvae (95% CI: 240–652) of *L. laticeps*, a tree with a dbh of 25 cm could provide pollen for 927 larvae (95% CI: 514–1429) of *B*. *terrestris* or 5202 larvae (95% CI: 2886–8022) of *L*. *laticeps*.

## DISCUSSION

In this study, we introduce an innovative method for estimating flower abundance on branches and trees by combining manual measurements with remote sensing data derived from TLS and QSMs. The key novelty of our approach lies in the two‐step upscaling process, which transitions from branch‐level to tree‐level models using remote sensing data, thereby eliminating the need for destructive sampling of entire trees or large crown sections. Additionally, we incorporated pollen production data to estimate the potential annual number of bee larvae that could theoretically be reared using the resources provided by cherry trees of varying sizes.

### Flowers at branch level

The models for predicting the probability of flower occurrence and the number of flowers differed slightly. The number of flowers per branch was additionally influenced by a positive interaction between dbh and the logarithm of branch diameter, meaning that branches on trees with a higher dbh were more strongly influenced by the nonlinear effect of branch diameter. Further, while tree and branch size were fundamental for flower abundance, flower probability seemed to be mainly determined by the position of the branch within the crown. This might indicate that the probability of flower occurrence and the number of flowers per branch result from different processes or conditions. Apart from these differences, the model of the probability of flower occurrence achieved a substantially worse fit than the model of flower abundance. This might indicate that we failed to survey some variables relevant to the process determining whether a branch bears flowers, or that this process might have a degree of randomness, as is common in nature (Mann, 1970), that cannot be modeled precisely.

The crown sections varied significantly in both the probability of flower occurrence and the number of flowers per branch, with branches in the outer and upper parts of the crown carrying more flowers than branches in the inner and lower parts. This finding confirms our expectation that branch position affects flower abundance. The crown sections in which branches tended to have higher flower probability and abundance are those that potentially have higher light availability. In accordance with this, a study by Tromp (1984) on apple trees showed that with reduced light intensity fewer flower buds are formed. Further, there is evidence that spurs develop primarily on branches with high light exposure, allowing flower bud development (Barritt et al., 1991).

Another factor likely influencing flower abundance on branches is the shoot type. As shown in studies by Yamashita (1971) and Fyhrie et al. (2018) on peach trees (*Prunus persica*), there are differences in flower bud density and flowering between different shoot types. Given these physiological differences, incorporating shoot types into the model could improve its predictive capabilities. Classifying different QSM branches into shoot types would require even more accurate modeling of branches but should be considered in future studies.

### Flowers at tree level

Our models show an exponential relationship between the number of flowers per tree based on dbh or crown volume. This confirms our expectation that the relationship between tree or crown dimension and flower abundance follows a nonlinear relationship. The nonlinearity can be illustrated using a simple factor: For a tree with dbh = 10 cm, increasing the dbh by x% leads to an increase in flower number by a factor of ${\mathrm{e}}^{0.01662x}$. Accordingly, a 100% increase to dbh = 20 cm leads to a 5‐fold increase in flowers, and a 150% increase to dbh = 25 cm leads to a 12‐fold increase in flowers.

The nonlinearity of the models is in line with other allometric functions regarding different tree traits, for example, volume and biomass (Picard et al., 2012). According to life history theory, organisms must allocate their resources to either growth, maintenance, or reproduction (Kaplan & Gangestad, 2015). While all three of these functions are critical, the available resources are limited and must therefore be distributed among these sinks. The distribution of resources varies between trees depending on endogenous and exogenous factors, such as life stage and environmental conditions. Larger, more mature trees tend to have more resources available but also invest an increasing fraction of their resources into reproduction (Genet et al., 2010; Thomas, 2011). Since tree dimension serves as a proxy for tree age, the increase in overall resources with increasing tree size, along with the increasing fraction of resources allocated for reproduction, could account for the nonlinear relationships that we observed. Since all trees face this tension between different resource sinks, we would generally also expect nonlinear relationships between tree dimension and floral abundance for other deciduous tree species, although the parameterisation of the model would most likely vary. Under extreme environmental conditions, where trees have almost no resources or have to invest large amounts of resources in maintenance due to external stressors, the relationship might be different. However, these hypotheses would need to be confirmed in future studies. It would also be interesting to investigate whether these results are transferrable to other growth forms apart from trees, for example, shrubs. Further, it would be interesting to know whether different management techniques which are known to alter reproductive growth, such as pruning or fertilization (Kozlowski & Pallardy, 1997), alter the shape of this relationship. Here, the pruning effect could not be investigated due to the lack of detailed information on the pruning of the investigated trees.

By all metrics of model performance, dbh outperformed crown volume as a predictor. This was surprising, as we had expected dbh to be a worse predictor. One reason could be that dbh is a better proxy for tree size, which is known to be related to reproductive growth (Thomas, 2011). Moreover, trees with higher dbh are likely dominant trees with higher light exposure and therefore sequester a higher total amount of carbon. In contrast, crown size and shape are the product of various factors such as competition and management. Pruning for high‐value timber production, which was conducted at our study site, typically involves the removal of branches in the lower crown to increase the length of the branch‐free bole (Balandier & Dupraz, 1999). This shifts the crown base height upward and reduces crown volume accordingly. As only a portion of the studied trees were pruned, this might have induced noise in the relationship between flower abundance and crown volume, negatively affecting the model fit. Therefore, we assume that crown volume might only have the potential to outperform dbh if the trees within an AFS are managed more similarly. However, confirming this would require further investigations in this direction.

Comparable to our study, Kay et al. (2020) estimated the number of flowers on cherry trees in AFS based on a method introduced by Baude et al. (2016). Flowers were counted in a representative section of the crown and the recorded flower density was scaled up to the whole tree based on crown volume. Kay et al. (2020) categorized their trees into three crown volume classes: 20, 105 and 352 m^3^. For these classes, they estimated trees to carry 0 to 50,000 flowers, 50,001 to 155,000 flowers, and 155,001 to 520,000 flowers, respectively. Our model predicts about 22,000 and 51,000 flowers for the first two classes. The third class is outside the calibration range of the model. Although in a similar range, our estimates are closer to the lower end of their estimates. This difference could be due to methodological differences. As shown by our models, flower abundance varies between different crown parts. Kay et al. (2020), however, sampled a single section in the crown and used this single flower density for upscaling on the whole tree level. Baude et al. (2016), whom Kay et al. (2020) followed, explicitly stated they sampled crown sections on the outer parts of the crown, where we found significantly higher floral abundance. The fact that our estimates were consistently lower than those of Kay et al. (2020) is also a confirmation that our estimates are unlikely to be overestimated by inflated branch diameters due to inaccuracies in the underlying TLS data. Besides methodological differences, differences could also be due to endogenous and exogenous factors differing between tree samples. These factors include tree age (Genet et al., 2010), genetics (Fyhrie et al., 2018; Lanauskas et al., 2023), alternate bearing (Butler, 1917; Lanauskas et al., 2023; Monselise & Goldschmidt, 1982), applied management techniques (Kozlowski & Pallardy, 1997; Kurlus et al., 2020; Stover, 2000) and environmental conditions (Kozlowski & Pallardy, 1997). To obtain a model that is able to reflect reality to a certain extent, our data cover some of the previously mentioned variability as we included trees of different ages and dimensions that were exposed to different pruning regimes. Nevertheless, it should be noted that the derived models are likely more reliable when applied to similar trees in terms of management and dimensions.

### Flowers at system level

As the nonlinearity of our models shows, large trees produce a disproportionately high number of flowers compared with smaller trees. However, the question is whether the nonlinearity still holds true when scaling up these results to entire AFS. This can be assessed by simulating different tree row setups using our models and additional allometric functions on crown dimensions of cherry trees in AFS (Schindler, Seifert, et al., 2023). For the simulation, we assume that the trees within a row (100 m) were planted at a distance ensuring the maximum number of trees without overlapping crowns at the given dbh values. A tree row containing only trees with a dbh of 10 cm could accommodate 34 trees, while a row with trees that have a dbh of 25 cm could accommodate only 14 trees. However, due to the nonlinearity of our model, tree rows containing 25‐cm trees are expected to produce five times as many flowers as those with 10 cm trees. Thus, the advantages of large trees regarding flower production and associated resources remain evident at the level of entire AFS. It should be mentioned, however, that the crown projection area in such an AFS would also be twice as large. Increased shading resulting from higher canopy closure could be beneficial for animal welfare (Pezzopane et al., 2019; Valtorta et al., 1997) and have neutral or positive effects on crop yields in some AFS (Lin et al., 1998; Somporn et al., 2012); however, the higher shading could also have negative effects on crop yields in other AFS (Dufour et al., 2013; Ehret et al., 2015).

### Flowers at landscape level

If our approach were applied to create similar models for other tree species that relate floral abundance to tree dimension, these small‐scale estimates of the number of flowers per tree could be extrapolated to the landscape level. Remote sensing techniques capable of collecting information at the landscape level, such as aerial imagery or airborne LiDAR, could be used to map and analyze trees at larger scales. The automatic delineation and species classification of trees using remote sensing data have been studied extensively (Fassnacht et al., 2016; Lindberg & Holmgren, 2017). Subsequently, tree canopy volume could be estimated, for example, using data from airborne LiDAR data (Zhu et al., 2021), to model floral resources for larger areas or even entire landscapes. By combining such spatial data with the flowering phenology of different tree species, new insights into the spatiotemporal distribution of floral resources, such as pollen and nectar, could be gained. This information could in turn help with the assessment and planning of agricultural landscapes in which pollinators need to be supported across the entire vegetation period to ensure crop pollination services.

### Foraging resources for bees

Our results show that a single wild cherry tree is able to provide enough pollen to rear several thousand wild bee larvae, depending on bee size. Establishing the connection between pollen supply per tree and the foraging resource requirements of bees can improve the planning of AFS to support pollination services. The nonlinearity of our models demonstrates that large trees produce a disproportionately high number of flowers, along with corresponding nectar and pollen, compared with smaller trees. Therefore, to promote bees and facilitate pollination services on an agricultural site, it would be more efficient to have a few large cherry trees rather than many small trees. Further, planting a large number of trees requires higher investments than planting fewer trees aiming at a smaller number of larger trees. Our findings highlight the disproportionate value of large trees, analogous to the (sometimes) disproportionate value of large land parcels in biodiversity conservation, which is relevant in the context of the SLOSS (*single large or several small*) debate in nature conservation (Le Roux et al., 2015; Yaynemsa, 2022).

When modeling the effects on bee populations and, consequently, pollination services based on the number of flowers per tree, several additional aspects that influence the visitation rates of bees and other pollinating insects should be considered. Flower density is not the only determining factor for pollinator populations; various other factors such as the diversity of floral resources (Hegland & Boeke, 2006; Venjakob et al., 2016), the phenological match between plants and pollinators (Roulston & Goodell, 2011), the availability of nesting sites and materials (Winfree, 2010), and competition or facilitation among pollinators (Brittain et al., 2013; Thomson & Page, 2020; Zimmerman & Pleasants, 1982) affect bee populations.

## CONCLUSIONS

A quantitative assessment of the resources provided by nature is essential for ecological and economic models that inform environmental and agricultural policy and law. Our approach can be utilized to develop models that predict floral abundance and associated resources such as pollen and nectar for trees of varying dimensions without the need for intensive destructive sampling. In this way, our study serves as a template for future research investigating further tree species and ecosystems. Besides flowers, leaf or fruit production can also be modeled using the same approach. Combining tree‐level resource models with the corresponding responses of insects or plants along gradients, such as tree dimension, may be used by the ecological community to model interacting organisms. This could enable a more comprehensive understanding of processes and drivers in agricultural and natural ecosystems. Besides ecological applications, this information could be further incorporated in decision‐making processes when planning subsidies for ecosystem service provision in AFS. For example, our results show the importance of growing and maintaining large trees, as they provide a disproportionately large amount of flower resources compared with smaller trees. This finding is likely applicable to other tree species and ecosystems and should, therefore, be considered when planning agricultural systems in which trees are planted with the goal of improving habitat suitability for pollinators and pollination services. Moreover, the finding that single large trees provide more resources for pollinators than several small trees covering the same area is likely applicable for other organisms, such as birds (which benefit from fruits for foraging and branches for nesting) or epiphytes (which utilize branch surfaces). Expressed in terms of the SLOSS debate, our study strongly supports the disproportionate value of “single large” entities for conservation.

## AUTHOR CONTRIBUTIONS

*Conceptualization*: Zoe Schindler, Christopher Morhart, and Thomas Seifert. *Methodology*: Zoe Schindler, Christopher Morhart, Thomas Seifert, Christian Vonderach, and Felix Fornoff. *Investigation*: Zoe Schindler, Christopher Morhart, and Felix Fornoff. *Formal analysis*: Zoe Schindler and Elena Larysch. *Visualization*: Zoe Schindler. *Writing—original draft preparation*: Zoe Schindler, Katja Kröner, and Nora Obladen. *Writing—review and editing*: Zoe Schindler, Christopher Morhart, Elena Larysch, Felix Fornoff, Katja Kröner, Nora Obladen, Alexandra‐Maria Klein, Thomas Seifert, and Christian Vonderach. *Funding acquisition*: Christopher Morhart, Thomas Seifert, Felix Fornoff, and Alexandra‐Maria Klein. *Supervision*: Christopher Morhart and Thomas Seifert.

## FUNDING INFORMATION

The project INTEGRA is supported by funds of the Federal Ministry of Food and Agriculture (BMEL) based on a decision of the parliament of the Federal Republic of Germany via the Federal Office for Agriculture and Food (BLE) under the Federal Programme for Ecological Farming and Other Forms of Sustainable Agriculture (support code 2819NA071).

## CONFLICT OF INTEREST STATEMENT

The authors declare no conflicts of interest.

## Supporting information


Appendix S1.

## Data Availability

Raw data and code (Schindler et al., 2025) are available in Zenodo at https://doi.org/10.5281/zenodo.15111778.
