# Supplementary material for: Flower power: Modeling floral resources of wild cherry (Prunus avium L.) for bee pollinators based on 3D data
Source: Ecology. 2025 May 8;106(5):e70103. doi: 10.1002/ecy.70103 (PMC12061797; doi:10.1002/ecy.70103)
Supplement: Supplementary file 1 — Appendix S1. [file ECY-106-e70103-s001.pdf]

Journal: Ecology

Title: Flower power: Modelling floral resources of wild cherry (*Prunus avium* L.) for bee pollinators based on 3D data

Authors: Zoe Schindler, Elena Larysch, Felix Fornoff, Katja Kröner, Nora Obladen, Alexandra-Maria Klein, Thomas Seifert, Christian Vonderach, Christopher Morhart

APPENDIX S1: SUPPLEMENTARY MATERIAL

Section S1: Estimating the number of larvae supported by floral resources of trees

As introduced by Müller et al. (2006), the average dry body mass of a single bee can be used to approximate the pollen volume of one brood cell, which is the required pollen volume necessary for rearing one larva. The equation describing this relationship is given in **Eq. S1**, where PV is the pollen volume of a single brood cell (mm³), BM is the dry body mass of a single bee (mg) and ε is the residual error. As a multitude of bee species forages on wild cherry and since pollen requirements vary with bee size, we wanted to calculate the pollen requirements for three wild bee species of varying size that commonly visit wild cherry: *Andrena helvola* L., *Bombus terrestris* L. and *Lasioglossum laticeps* Schenk. Additionally, we wanted to calculate the pollen requirements of the commercially used *Apis mellifera* L. As we did not have the dry body weight of these species, we constructed a regression relating body length BL (mm) to dry body mass BM (mg) based on the data from Müller et al. (2006). The regression achieved a good model fit with an adjusted R^2 of 0.87 and is given in **Eq. S2**. Using body lengths reported by Westrich (2019), the dry body mass and subsequently the pollen requirements of the selected species were estimated: *An. helvola* (*BL* 11–12 mm, *BM* 22.62 mg, *PV* 39.62 mm³), *Ap. mellifera* (*BL* 11–13 mm, *BM* 25.28 mg, *PV* 43.59 mm³), *B. terrestris* (*BL* 11–17 mm, *BM* 37.83 mg, *PV* 61.65 mm³), *L. laticeps* (*BL* 6–7 mm, *BM* 5.09 mg, *PV* 10.98 mm³). Using the pollen volume per flower, flower abundance per tree and the species-specific pollen requirements, we calculated the number of larvae that can theoretically be reared using the resources from trees of varying dimensions.

$$\log(PV) = 0.433 + 0.868 \times \log(BM) + \varepsilon \qquad (Eq.\,S1)$$

$$\ln(BM) = -3.265 + 2.614 \times \ln(BL) + \varepsilon \qquad (Eq.\,S2)$$

References

Müller A, Diener S, Schnyder S, Stutz K, Sedivy C, Dorn S (2006) Quantitative pollen requirements of solitary bees: Implications for bee conservation and the evolution of bee–flower relationships. Biological Conservation 130:604–615. https://doi.org/10.1016/j.biocon.2006.01.023

Westrich P (2019) Die Wildbienen Deutschlands. Verlag Eugen Ulmer Stuttgart

SUPPLEMENTARY FIGURES

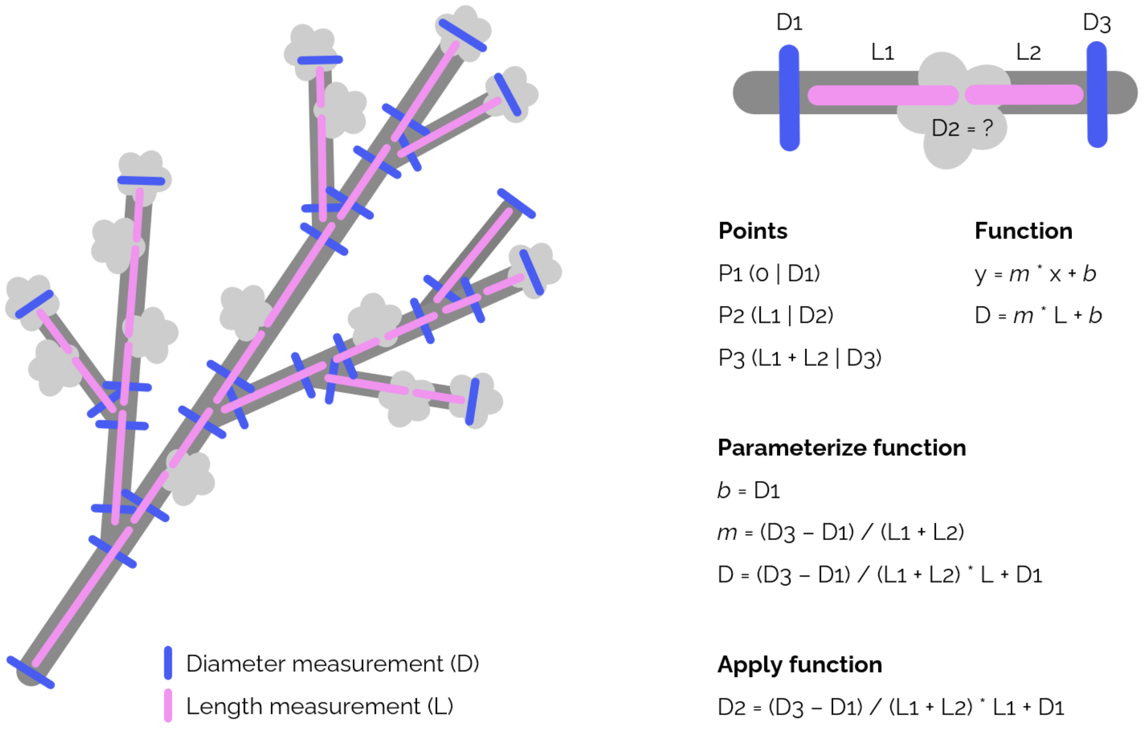

Fig. S1 Visualisation of the reconstruction of branch diameters to which flowers were attached. The calculation of the branch diameter a flower was attached to was based on the assumption of linear diameter change between the consecutive diameter measurements, i.e. using simple linear models. For each non-bifurcated segment, a linear function was parameterised using the respective diameter and length measurements. This function was then used to identify both the branch diameters flowers were attached to and to calculate at which lengths there was a switch in diameter classes, as defined as 5 mm bins (i.e. 0–5 mm, 5–10 mm, etc.). These data were then combined to calculate the number of flowers within diameter classes, the cumulated branch length within diameter classes, and, as a result, the number of flowers per branch length within diameter classes. Credit: Zoe Schindler.

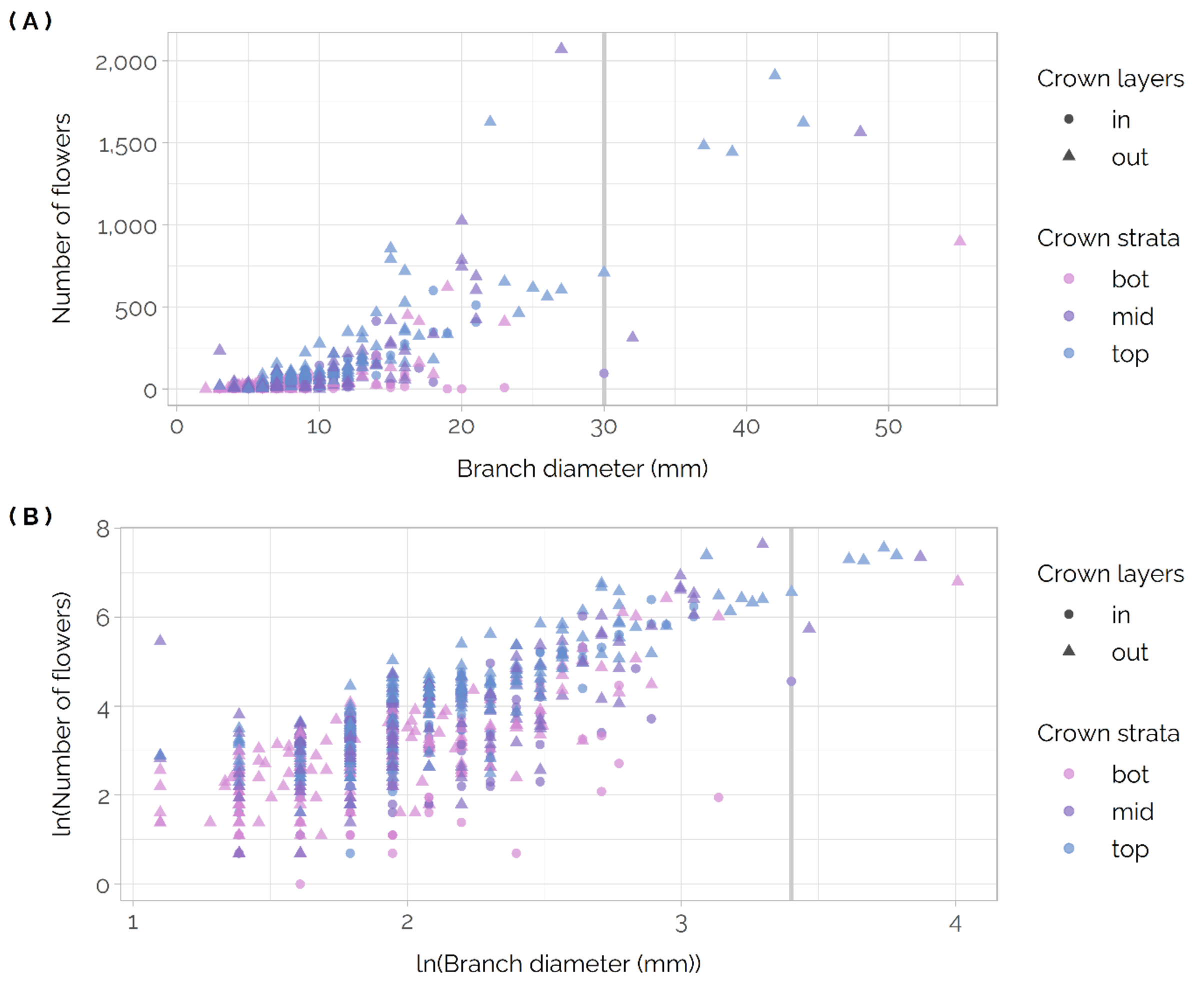

Fig. S2 Number of flowers per branch, including the flowers on the respective sub-branches, before the sub-sampling. The crown layers are indicated by the point shape, while the crown strata are indicated by point color. The vertical lines indicate the diameter which was used as the maximum branch diameter for upscaling the number of flowers per branch to whole trees, i.e. 30 mm. **(A)** Original data. **(B)** Log-transformed data.

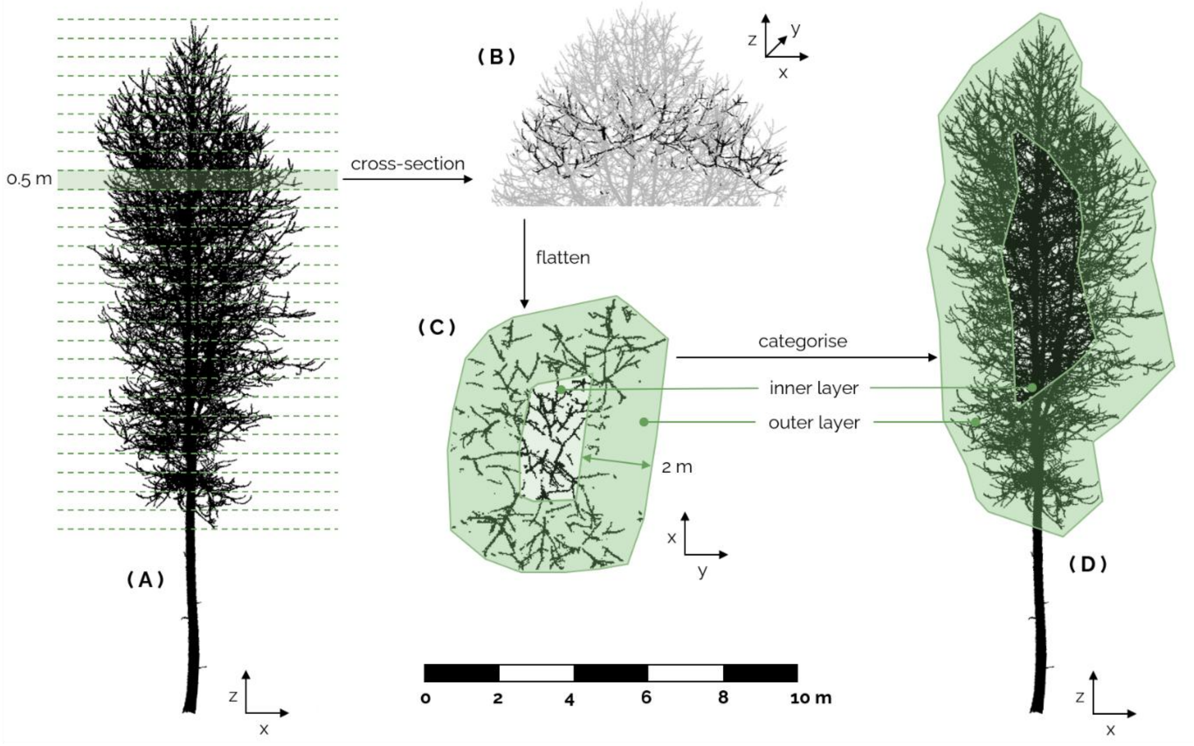

Fig. S3 Diagram showing the workflow of the classification of the crown layers. In the paper, the centres of the QSM cylinders were used instead of the raw point cloud, but for better visibility, we used the denser raw point cloud for this visualisation. **(A)** The tree crown is separated into 0.5 m high vertical cross-sections; the selected section is highlighted in green. **(B)** The cross-section is shown in black, with the remaining points shown in grey. **(C)** The cross-section is projected to the xy-plane and a convex hull around the points is calculated (green polygons) and buffered by 2 m. The points within the buffer (dark green polygon) belong to the outer crown, while points interior to the buffer (light green polygon) belong to the inner crown. **(D)** Result of the classification after repeating the categorisation for all cross-sections. Credit: Zoe Schindler.

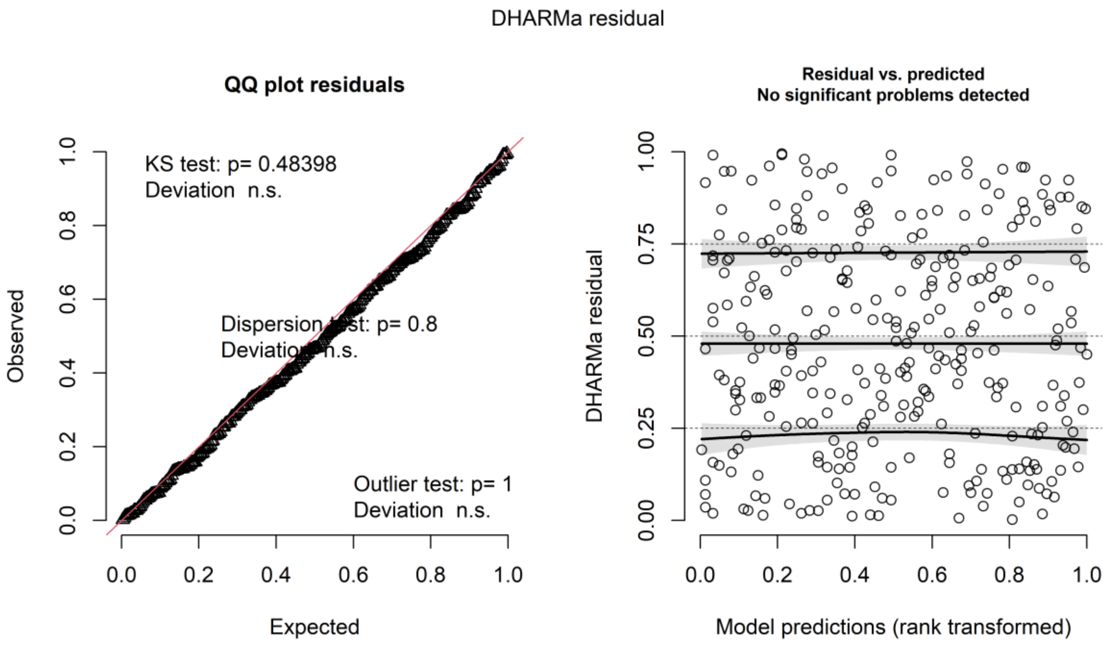

Fig. S4 Diagnostic plots from the *DHARMa* package for the branch-level model on flower probability.

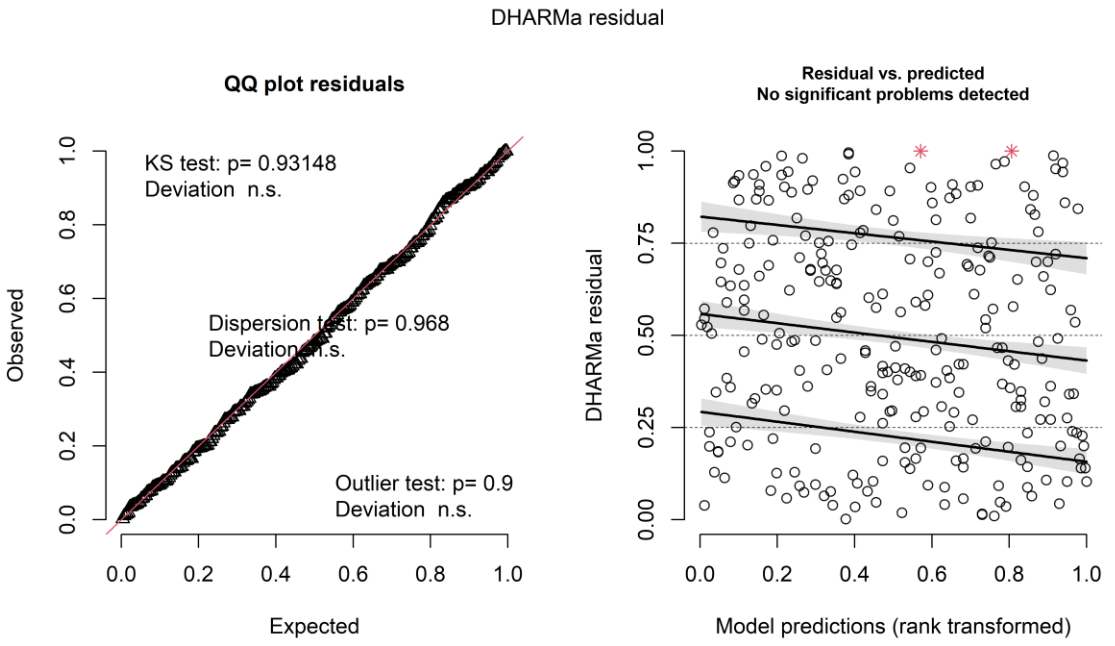

Fig. S5 Diagnostic plots from the *DHARMa* package for the branch-level model on the number of flowers.

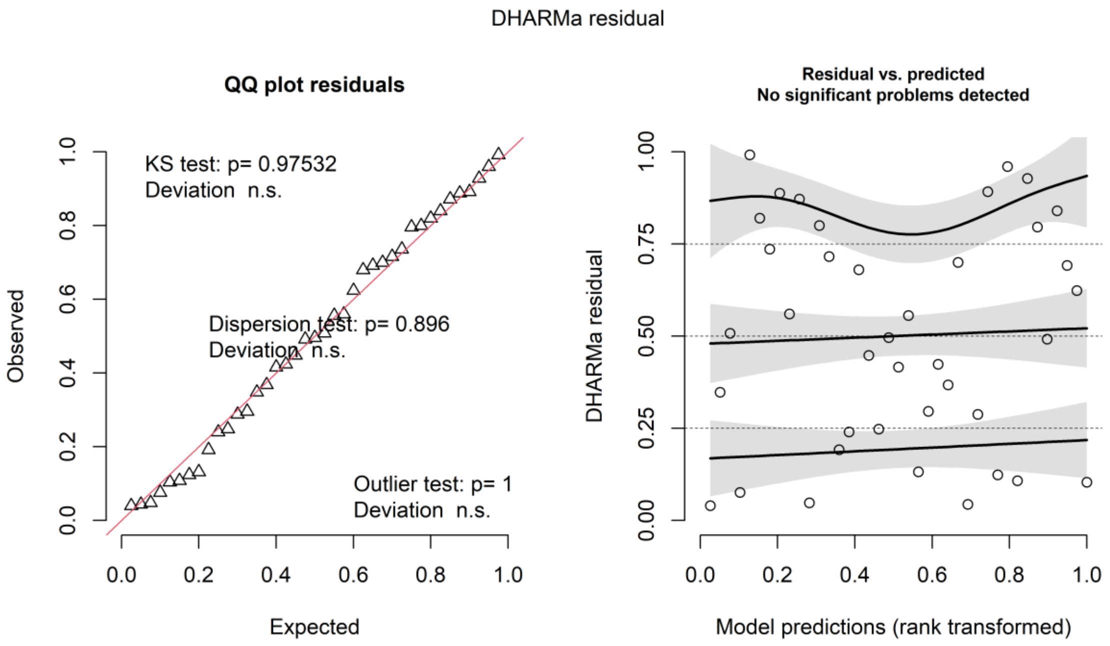

Fig. S6 Diagnostic plots from the *DHARMa* package for the tree-level model on the number of flowers based on DBH.

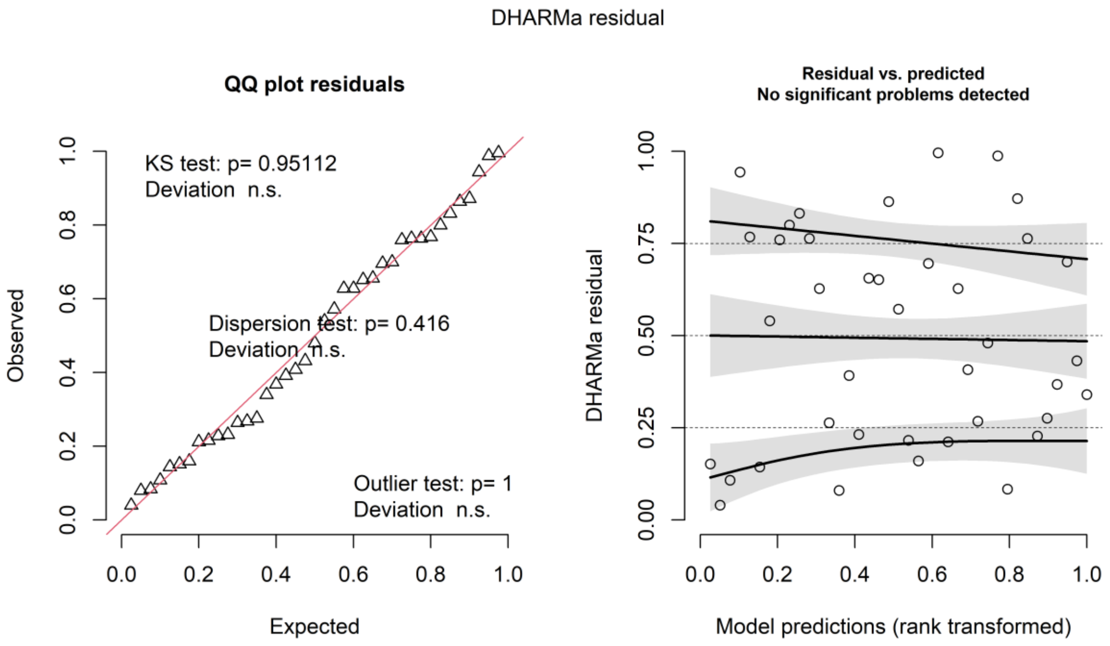

Fig. S7 Diagnostic plots from the *DHARMa* package for the tree-level model on the number of flowers based on crown volume.

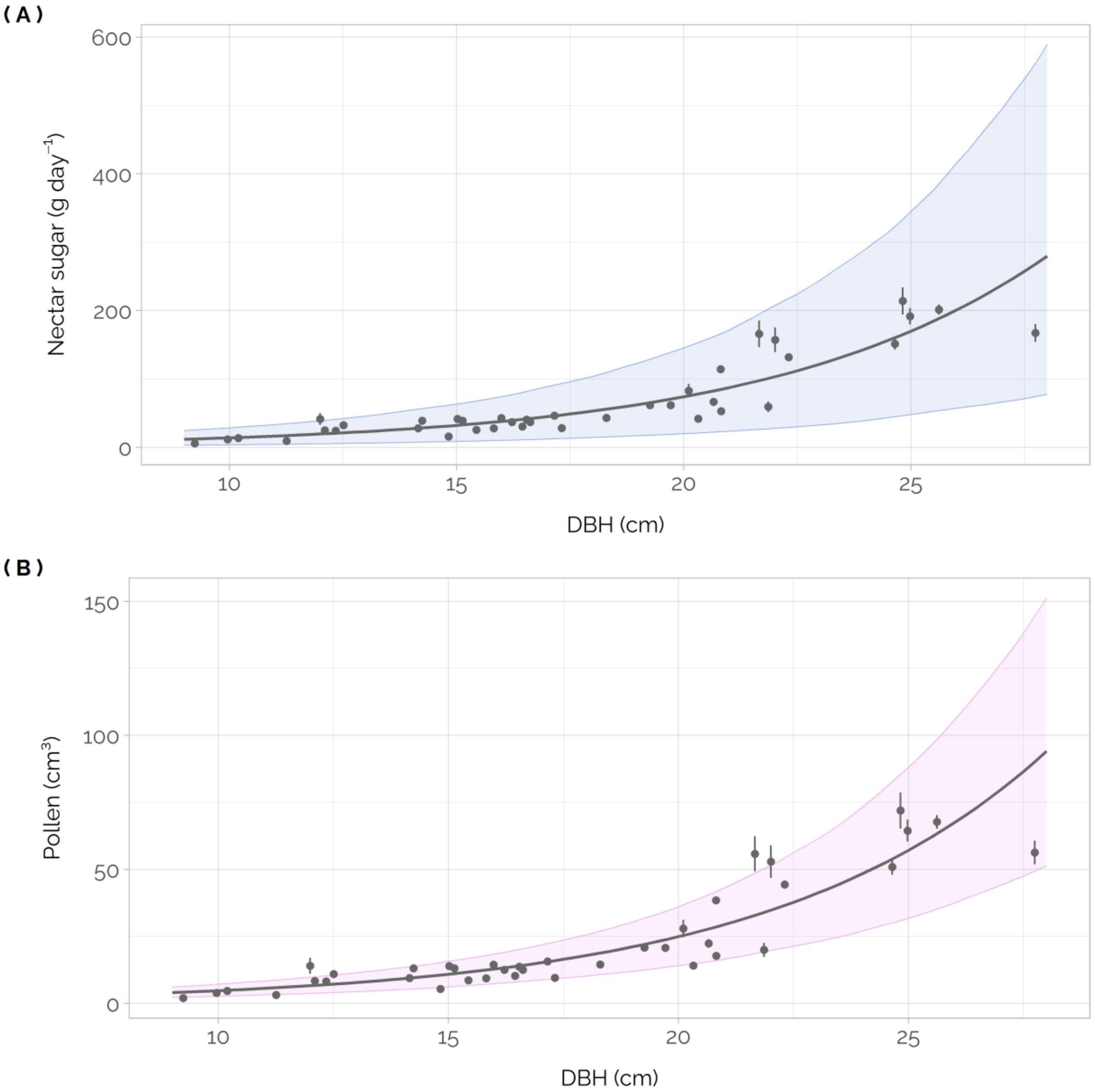

Fig. S8 Estimates on the **(A)** daily nectar sugar production (g day^{-1}) and **(B)** the provided pollen volume (cm^3) per tree based on DBH (diameter at breast height, cm). The shaded areas indicate the 95% confidence intervals of the estimates, derived from 1,000-fold bootstrapping of the model estimating the number of flowers per tree and Monte Carlo simulations on the parameters required for estimating these resources from the number of flowers per tree. The error bars show the uncertainty of the QSM (quantitative structure model) reconstruction with median ± SD for the number of flowers combined with the average nectar sugar and pollen production per flower.

SUPPLEMENTARY TABLES

Table S1 Results of the asymptotic one-sample Kolmogorov-Smirnov test (*DHARMa::testUniformity*). The table gives the test statistic and the p-values for the branch- and tree-level models.

Model	D-value	p-value
Branch		
Flower occurrence	0.0477	0.4840
Flower count	0.0331	0.9315
Tree		
Flower count, DBH	0.0731	0.9753
Flower count, crown volume	0.0830	0.9511

Table S2 Results of the DHARMa bootstrapped outlier test (*DHARMa::testOutliers*). The table gives the test statistic and the p-values for the branch- and tree-level models.

Model	outliers	observations	p-value
Branch			
Flower occurrence	0	308	1
Flower count	2	268	1
Tree			
Flower count, DBH	0	39	1
Flower count, crown volume	0	39	1

Table S3 Results of the DHARMa nonparametric dispersion test via SD of residuals fitted vs. simulated (*DHARMa::testDispersion*). The table gives the test statistic and the p-values for the branch- and tree-level models.

Model	dispersion	p-value
Branch		
Flower occurrence	1.0276	0.8000
Flower count	0.6172	0.9680
Tree		
Flower count, DBH	0.8349	0.8960
Flower count, crown volume	0.5693	0.4160

Table S4 Results of the test for location of quantiles via qgam (*DHARMa::testQuantiles*). The table gives the test statistic and the p-values for the branch- and tree-level models.

Model	p-value
Branch	
Flower occurrence	0.6626
Flower count	0.1246
Tree	
Flower count, DBH	0.3365
Flower count, crown volume	0.9344

Table S5 Estimated marginal means (*emmeans::emmeans*) for the different crown strata (bot, mid, top) and crown layers (in, out) based on the branch-level models of the probability of flower occurrence and of the flower counts.

Model	Level	Contrast	(odds-) ratio	SE	df	null	z-ratio	p-value
Flower occurrence	bot	out / in	29.40	21.70	Inf	1	4.571	< 0.0001
	mid	out / in	0.62	0.50	Inf	1	- 0.591	0.5548
	top	out / in	13.79	16.10	Inf	1	2.250	0.0244
Flower occurrence	out	bot / mid	4.68	3.60	Inf	1	2.002	0.1118
		bot / top	1.16	1.43	Inf	1	0.120	0.9922
		mid / top	0.25	0.29	Inf	1	- 1.201	0.4524
	in	bot / mid	0.10	0.08	Inf	1	- 3.039	0.0067
		bot / top	0.54	0.37	Inf	1	- 0.889	0.6473
		mid / top	5.51	4.67	Inf	1	2.014	0.1088
Flower count	bot	out / in	3.26	0.76	Inf	1	5.052	< 0.0001
	mid	out / in	2.31	0.47	Inf	1	4.122	< 0.0001
	top	out / in	1.76	0.44	Inf	1	2.268	0.0233
Flower count	out	bot / mid	0.58	0.11	Inf	1	- 2.942	0.0091
		bot / top	0.41	0.09	Inf	1	- 3.973	0.0002
		mid / top	0.70	0.15	Inf	1	- 1.653	0.2237
	in	bot / mid	0.41	0.09	Inf	1	- 3.876	0.0003
		bot / top	0.22	0.06	Inf	1	- 5.835	< 0.0001
		mid / top	0.54	0.11	Inf	1	- 2.954	0.0088
